# Overcoming the Limits of Flash Nanoprecipitation: Effective Loading of Hydrophilic Drug into Polymeric Nanoparticles with Controlled Structure

**DOI:** 10.3390/polym10101092

**Published:** 2018-10-02

**Authors:** Daniele Massella, Edvige Celasco, Fabien Salaün, Ada Ferri, Antonello A. Barresi

**Affiliations:** 1 Department of Applied Science and Technology, Politecnico di Torino, Corso Duca degli Abruzzi 24, 10129 Torino (TO), Italy; ada.ferri@polito.it (A.F.); antonello.barresi@polito.it (A.A.B.); 2 ENSAIT, GEMTEX—Laboratoire de Génie et Matériaux Textiles, F-59000 Lille, France; fabien.salaun@ensait.fr; 3 College of Textile and Clothing Engineering, Soochow University, Suzhou 215123, China; 4 Dipartimento di Fisica dell’Università degli studi di Genova, Via Dodecaneso 33, 16146 Genova (GE), Italy; celasco@fisica.unige.it

**Keywords:** nanoparticles, caffeine, flash nanoprecipitation, solvent displacement, drug delivery, PCL, CIJM, encapsulation efficiency, hydrophilic compound, surface properties

## Abstract

Flash nanoprecipitation (FNP) is a widely used technique to prepare particulate carriers based on various polymers, and it was proven to be a promising technology for the industrial production of drug loaded nanoparticles. However, up to now, only its application to hydrophobic compounds has been deeply studied and the encapsulation of some strongly hydrophilic compounds, such as caffeine, remains a challenge. Caffeine loaded poly-ε-caprolactone (PCL) nanoparticles were produced in a confined impinging jet mixer using acetone as the solvent and water as the antisolvent. Caffeine was dissolved either in acetone or in water to assess the effects of two different process conditions. Nanoparticles properties were assessed in terms of loading capacity (LC%), encapsulation efficiency (EE%), and in vitro release kinetics. Samples were further characterized by dynamic light scattering, scanning electron microscopy, X-ray photo electron spectroscopy, and infrared spectroscopy to determine the size, morphology, and structure of nanoparticles. FNP was proved an effective technique for entrapping caffeine in PCL and to control its release behavior. The solvent used to solubilize caffeine influences the final structure of the obtained particles. It was observed that the active principle was preferentially adsorbed at the surface when using acetone, while with water, it was embedded in the matrix structure. The present research highlights the possibility of extending the range of applications of FNP to hydrophilic molecules.

## 1. Introduction

The production of nanocarriers is one the most innovative and promising research fields in modern pharmaceutical technology: As a matter of fact, the use of nanoscale formulations can improve drug compatibility [1,2], provide more accurate diagnosis [3,4], reduce the risk of immune response [5,6], and achieve targeted drug delivery [7,8]. Despite the aforementioned scientific advancements, the market of nanoscale medical devices is still a niche one, not only because of regulatory issues, but also because of the difficulties encountered in producing nanocarriers at an industrial scale [9]. Concerning the scale up of polymeric nanoparticles production technologies, flash nanoprecipitation (FNP) technique has attracted significant interest, mainly because of its ability to combine simplicity and rapidity with good product quality, stability, and control of particle size distribution (PSD) [10]. The formation of particles is obtained by mixing a water-miscible organic solvent, in which a preformed polymer is solubilized, with a water (or anti-solvent) phase, which allows instant precipitation of the polymer [11]. The governing factor in the nanoparticle formation is mixing [12]; therefore, special micro-mixers have been designed and tested to effectively control mixing efficiency [13].

Among these devices, the confined impinging jet mixer (CIJM) has been widely used in several experimental works [14,15]. The solvent and the antisolvent streams are pumped through the arms of the device and the jets collide inside the mixing chamber; the strong turbulence allows intensive mixing, and the rapid generation of local supersaturation in the polymer solution causes polymer precipitation in the form of nanoparticles [15,16,17]. The justification of the chemico-physical phenomena occurring in the CIJM is further provided by CFD modelling studies [18,19,20], which contributes to make FNP a robust and well known process. The flash nanoprecipitation technique has been mainly used to entrap highly hydrophobic drugs (log*P* > 3.5) to achieve encapsulation efficiencies close to 100%. Such high yields are due to the scarce affinity of hydrophobic drugs with water and the concurrent precipitation of the drug and polymer upon jet collision [21,22,23]. Obviously, the encapsulation of hydrophilic drugs by FNP is much more difficult if water is used as the antisolvent, and this is actually considered the major drawback of this technology [10,24]. Therefore, during the last two years, a few studies were conducted to assess the feasibility of encapsulating hydrophilic substances by FNP. It was shown that the range of application of FNP could be extended to lowly hydrophobic substances (log*P* = 1–2) even if lower encapsulation efficiencies were achieved (50–80%) [25,26]. Furthermore, the method proposed by Allen [27] greatly expanded the capabilities of FNP by simultaneously loading hydrophilic and hydrophobic substances, as hydrophilic compounds tend to interact with the nanocarriers’ surface; with strongly hydrophilic substances, the maximum achieved EE% was lower than 20%. To our knowledge, studies concerning FNP performance in encapsulating strongly hydrophilic compounds, such as caffeine (log*P* = −0.07) [28], have not been extensively conducted. In the present work, caffeine was selected for encapsulation in PCL nanoparticles via FNP to prove the limit of this technique and obtain insight into the mechanisms of partitioning the hydrophilic drug between the polymer and the liquor.

Caffeine (CAF) is a methylxanthine and a naturally alkaloid found in a wide variety of plants, beans, and leaves [29]. Its large application in food, pharmaceuticals, cosmetics, and supplements makes caffeine one of the most consumed drugs worldwide [30]. Caffeine is a well-known mild stimulant of the central nervous system; it increases mental alertness and concentration and reduces mental fatigue [31]. Moreover, it is used as a dietary supplement in sports activity to promote physical performance and ease weight loss for cosmetic purposes [29,32]. This drug is also suitable for skin protection from UV-light damage, and thus plays a key role in the prevention and cure of skin cancer [33,34]. Moreover, caffeine promotes cellular lipolysis and acts as an anti-cellulite drug in skin topical applications [35]. Upon oral administration, caffeine is adsorbed quickly into the bloodstream, its clearance into the stomach takes only 20 min and its concentration peak in the blood is reached after about 1 h; once in the blood, caffeine circulates throughout the body, ending up in the liver where it is metabolized [36]. The delivery of caffeine to the circulatory system is mainly due to its marked hydrophilicity that allows easy dissolution in aqueous media, such as human blood [37]. Nevertheless, this latter characteristic causes oral administration to be uncontrolled while delivery through the skin is not effective because of the lipophilic character of the skin outer layers [38]. To overcome this drawback, caffeine has been successfully encapsulated using various nanocarriers, such as liposomes [39,40], polymeric nanoparticles [41,42], and nanostructured lipid carriers [43,44]. However, scientific literature has also highlighted that caffeine encapsulation efficiency higher than 30% is rarely achieved using the above mentioned carriers, whichever the encapsulation method adopted. Thus, caffeine encapsulation is a challenging issue and the encapsulation efficiency (EE) benchmark for evaluating its encapsulations should range between 20% and 30%.

The aim of this study is to determine whether, and under which conditions, the FNP technique may be used to encapsulate a hydrophilic substance; for this reason, caffeine was selected as the model drug to be incorporated in poly-ε-caprolactone (PCL), a biocompatible and biodegradable polymer, widely applied in tissue engineering, implantable devices, cell cultures, and drug delivery [45,46,47]. Caffeine was either solubilized in acetone, the polymer solvent, or in water, the anti-solvent, prior to being injected into a CIJM to produce nanoparticles. The process parameters (flow rates) as well as the formulations (caffeine solvent, concentration, caffeine to polymer mass ratio) were investigated to understand their role and clarify under which conditions caffeine encapsulation can be achieved. Given the interest in dermatological applications, the formulations that displayed suitable characteristics for transdermal release were studied in more depth. The tested formulations were characterized by determining their encapsulation efficiency (EE) and loading capacity (LC) by UV spectroscopy to compare the effectiveness of FNP with other caffeine encapsulation techniques. Particular attention was paid to characterize the effect of the solvent chosen for caffeine on the particles’ structure by zeta potential (Zp), X-ray photoelectron spectroscopy (XPS), and scanning electron microscopy (SEM) analysis. Furthermore, the in vitro drug-release behavior of the nanocarriers was monitored by a dialysis method to establish the predominant drug release kinetics.

## 2. Materials and Methods

### 2.1. Materials

All chemicals were purchased from Sigma Aldrich (Milan, Italy). Caffeine in anhydrous powder form ReagentPlus Grade and PCL preformed in flakes with an average molecular weight of 14,000 g/mol were used. Acetone with purity ≥ 99.5% in compliance with European Pharmacopedia standards and acetonitrile of an HPLC grade ≥ 99.9% were employed as solvents. Phosphate buffer solution was prepared with the following chemicals: 8 mg/mL sodium chloride anhydrous ≥ 99%, 0.2 mg/mL potassium chloride ACS grade ≥ 99.5%, 1.44 mg/mL sodium phosphate dibasic dehydrate ≥ 99%, and 0.24 mg/mL potassium dihydrogen phosphate ACS reagent ≥ 99%. Ultrapure water was produced by means of a Milli-Q RG system by Millipore R (Billerica, MA, USA).

### 2.2. Nanoparticle Preparation

The preparation of the nanoparticles was carried out in a CIJM (1 mm inlet tube diameter, 5 mm chamber diameter, 11.2 mm chamber height, and a 2 mm exit tube, 40 mm long). A stream of PCL in acetone solution (Solution 1) was mixed with an aqueous stream used as the anti-solvent (Solution 2).

The polymer was in preformed flakes so that the molecular weight was kept constant during the precipitation stage. The collision of the two jets induced polymer precipitation in the form of nanoparticles. To prepare Solution 1, PCL was dissolved in acetone with a concentration ranging from 4.5 to 15 mg/mL. Caffeine was dissolved either in the solvent Solution 1 or in the water Solution 2 at various concentrations to obtain different caffeine to polymer mass ratios (MR).

The two solutions were placed in two 100 mL syringes and pumped to the CIJM through plastic tubes by means of a syringe pump (KDS200, KD Scientific, Holliston, MA, USA). The flow rate was generally kept constant at 20 mL/min since dynamic ligth scatter (DLS) analysis showed a mean diameter smaller than 450 nm, which is the threshold for dermatological applications, but a few runs were carried out at higher flow rates (up to 80 mL/min) to confirm the effect of flow rate on particle size. Samples of 6 mL (3 mL for each stream) were taken, and were collected in a glass vial containing 3 mL of quenching water placed downstream of the mixer to avoid further growth of the particle size. The quenched samples were kept under magnetic stirring for two minutes and then used for further characterizations.

### 2.3. Analytical Methods

#### 2.3.1. Nanoparticle Size

Particles size distribution, mean diameter, and polydispersity index were measured by means of DLS Zetasizer Nanoseries ZS90, Malvern Instruments (Malvern, UK). Samples were prepared by diluting 0.1 mL of the nanoparticle suspensions in 1 mL of ultrapure water. All samples were measured in triplicate under a controlled temperature of 25.0 ± 0.1 °C.

#### 2.3.2. Determination of Loading Capacity and Encapsulation Efficiency

LC is defined as the mass of the encapsulated drug (*m*_en_) divided by the mass of the whole polymeric nanoparticles system (*m*_tot_), as given by Equation (1); it is an index of the amount of drug that can be incorporated in a given amount of nanoparticle formulation. EE is defined as the amount of encapsulated caffeine over its input quantity in the process (*m*_in_), as expressed by Equation (2); it is an index of the efficiency of the nanoparticles’ production process.

(1) $$\;\mathrm{LC}\left(\%\right)=\frac{m_{en}}{m_{tot}}\times 100\;$$

(2) $$\;\mathrm{EE}\left(\%\right)=\frac{m_{en}}{m_{in}}\times 100\;$$

To calculate LC% and EE%, the methodology described in [26] with slight modifications was employed. The formed nanoparticles were carefully separated from the liquid phase by an experimental procedure consisting of several steps. Firstly, the nanoparticles suspension was placed in a rotary evaporator, RE 300 (Buchi, Switzerland), at 45 °C, under vacuum for 15 min, to remove acetone. Then, water was refilled to the suspension up to the initial volume and the nanoparticles were separated from the liquid phase by centrifugation for 1 h at 15,777× *g* in an SL 16 centrifuge by Thermo Scientific (Langenselbold, Germany). The excess supernatant was removed from the particles by a pipette. We estimated an LC maximum error of 1% due to caffeine contained in the supernatant adhering to wet nanoparticles. A 0.1 mL supernatant sample was diluted in 25 mL of water and filtered with a 0.2 µm cellulose syringe filter and then analyzed by UV-Vis spectroscopy using a 6850 UV/Vis Jenway spectrophotometer (Stone, Staffordshire, UK). The precipitated nanoparticles were dried overnight at 40 °C and weighted. Throughout all the described procedures, DLS measurements were taken to check whether PSD had significantly changed. The calculation of LC% and EE% was performed both by a ‘direct’ and ‘indirect’ method to double check the reliability of the analysis. In the indirect method, EE% and LC% were calculated by determination of free caffeine in the filtered supernatant. The filtered supernatant was analyzed by UV-visible spectrophotometry and the absorbance peak at the wavelength of 273 nm was considered for caffeine quantification. The supernatant of an unloaded PCL suspension was used as a blank in the spectrophotometric measurement to eliminate the interference of unfiltered suspended nanoparticles (smaller than 0.2 μm) in the supernatant due to the Tyndall effect. In the direct method, the LC% and EE% were calculated by dissolving a weighted amount of dry nanoparticles in acetonitrile and analyzing the caffeine absorbance in the resulting solution. This method allowed the amount of polymer to be estimated gravimetrically in which caffeine was contained, without relying on the assumption that all PCL was precipitated in the nanoparticle form (as assumed in the indirect method). To verify the quantification procedure, the caffeine mass balance was checked by summing up the amount in the supernatant and the one in the dry nanoparticles. In the case of recovered caffeine being lower than 97%, the data were considered unreliable and discarded. A list of the formulations for which loading capacity and encapsulation efficiency have been evaluated is given in Table 1.

#### 2.3.3. Fourier Transform Infrared Spectroscopy

The chemical structure of the nanoparticles was analyzed by FT-IR spectroscopy in the attenuated total reflectance mode. Samples of the dried powder were placed on a ZnSe crystal, and ATR-FTIR spectra in the absorbance mode were recorded using a Nicolet Nexus (Thermo Fisher Scientific, Villebon sur Yvette, France), connected to a PC (computer), in which the number of scans was 254 and the resolution was 1 cm^−1^.

#### 2.3.4. Zeta Potential

Zeta potential measurements were performed by DLS Zetasizer Nanoseries ZS90 (Malvern Instrument, Malvern, UK). Before measurement, acetone was removed in the rotary evaporator and the suspension was diluted 1:10. Temperature was controlled at 25 ± 0.1 °C during the measurement. Samples with MR varying in the range 0.75–1.50 have been considered. A list of the formulations for which the zeta potential was measured is given in Table 2.

#### 2.3.5. X-ray Photoelectron Spectroscopy

The nanoparticle surface composition was analyzed by XPS Versa Probe 5000, PHI Electronic (Chanhassen, MN, USA). The system is equipped with dual charging neutralization guns. They consist of an electron gun combined with an argon ion gun, at low energy, for minimizing the charging effect during data acquisition. The same argon gun was employed during the depth profile analyses, which was conducted in the etching mode, at higher energy, achieving an etching rate of 5.9 nm/min. This etching procedure was performed simultaneously to the scanning analyses, providing a real time chemical information of the first external layers of the nanoparticles’ structures.

#### 2.3.6. Field Emission Scanning Electron Microscopy

The nanoparticle morphology was observed by means of field emission scanning electron microscopy (FESEM) Zeiss Merlin (Oberkochen, Germany) with an acceleration voltage of 5 kV and a current probe of 200 pA. The samples were placed on aluminum specimen stubs and sputter-coated with chromium to reduce the charging effect during the analyses.

#### 2.3.7. In Vitro Release Test

In vitro release tests were performed by the dialysis method, using a pH = 7.4 phosphate buffer solution (PBS) as the receptor medium. Each release sample was prepared as follows: Three batches of NP suspensions were produced and centrifuged, then 7 mL of supernatant volume was removed from each batch and analyzed in terms of the LC% and EE%. 2 mL suspensions from each batch were mixed inside the dialysis tube (MW cut-off 12000 Da, Sigma Aldrich) and sank inside the receptor fluid to make a single release test. For each formulation, three release tests were run in parallel and the cumulative release curve was given in terms of the mean and standard deviation. The receptor fluid was kept under gentle stirring, 5 mL liquid samples were withdrawn periodically, and the withdrawn volume was replaced by fresh PBS solution. The caffeine concentration was determined by UV spectrophotometry. Caffeine cumulative release data were collected and fitted by 5 models, i.e., (i) Zero-Order; (ii) First-Order; (iii) Higuchi; (iv) Hixon-Crowell; and (v) Baker-Lonsdale model [48,49]. The regressions were carried out by interpolating the data until the asymptote in the cumulative release curve was reached. The contribution of free caffeine to the released curves was calculated by subtracting the free caffeine curve from the NP curves before regression. The model with the highest correlation coefficient, *R*^2^, was considered to be the best fit for the release kinetics. The zero-order model describes a system with a drug release rate independent of its concentration (Equation (3)). The data were plotted versus time.

(3) $$\;Q-Q_{0}=K_{0}t\;$$

where *Q* and *Q*_0_ are the quantities of released caffeine at time *t* and *t*_0_, respectively, and *K*_0_ (time^−1^), the zero-order release constant. In the first-order model, the release of drug was expressed by Equation (4):

(4) $$\;\log Q=\log Q_{0}-\frac{Kt}{2.303}\;$$

where *K* is the first-order rate constant expressed in units of time^−1^ and *t* is the time.

The Higuchi model was used to describe caffeine release from the nanoparticle as a square root of the time dependent process based on the Fickian diffusion model (Equation (5)):

(5) $$\;Q=K_{H}\sqrt{t}\;$$

where *K*_H_ is the Higuchi release constant.

The Baker and Lonsdale’s model was used to describe controlled drug release from a spherical matrix (Equation (6)):

(6) $$\;\frac{3}{2}\left[1-{\left(1-\frac{Q}{Q_{\infty }}\right)}^{\frac{2}{3}}\right]\times \frac{Q}{Q_{\infty }}=Kt\;$$

where *K* is the release constant, corresponding to the angular coefficient of the curve. *Q*/*Q*_∞_ is the percentage of released caffeine, with respect to the plateau, at time *t*.

The Hixson-Crowell model described the release from systems where there is a change in the surface area and diameter of the particles (Equation (7)); this would be applicable, for example, if the release rate of caffeine was controlled by an erosion, dissolution, and degradation mechanism of the PCL particles.

(7) $$\;\sqrt[{3}]{Q_{0}}-\sqrt[{3}]{Q}=K_{\mathrm{HC}}\;$$

where *K*_HC_ is the release constant of the model.

## 3. Results and Discussions

### 3.1. Formation of PCL-caffeine Nanoparticles

**Figure 1:**
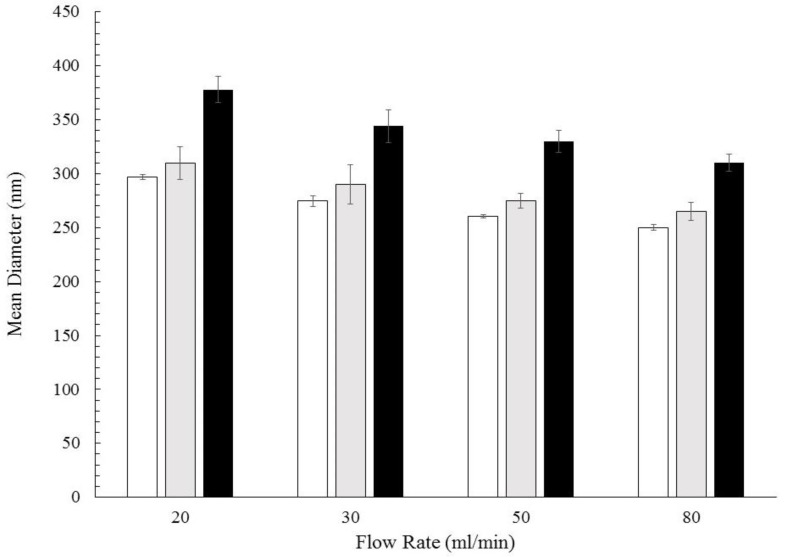
Size of PCL-caffeine nanoparticles obtained at different flow rates with different formulations: White bar: PCL 6 mg/mL; grey bar: PCL-CAF_1.5_-W; black bar: PCL-CAF_1.5_-Ac.

The manufacture of the nanoparticles was carried out by flash nanoprecipitation, a solvent-displacement method, which allows the production of drug loaded nanoparticles with a narrow size distribution. In this study, the PCL macromolecules were dissolved in acetone, a so-called “good” solvent, and then mixed with water, a “bad” solvent for PCL, but fully miscible with acetone. The presence of water induces the macromolecules’ aggregation in clusters or nanoparticles. The particle formation mechanism can be very complex due the possible simultaneous presence of several phenomena, such as nucleation, self-assembling, growth, and aggregation [11,50]; different simplified mechanisms have been proposed, however, recent results evidenced that the controlling step in particle formation can change with the operating conditions and species concentrations [19,51,52]. The extent of each of these phenomena determines the characteristics of the produced NPs. The dominance of nucleation leads to a larger number of particles with a smaller size, while when the growth phenomena prevail (by molecular growth, assembling, or aggregation), larger particles can be obtained. Mixing and supersaturation conditions inside the reactor chamber determine the prevailing mechanisms and are the key factors influencing the particle size and structure. The mixing conditions and the turbulence level can be controlled by tuning the inlet flow rate to CIJM. However, their effect is complex because they affect both the build-up of the supersaturation and the growth mechanisms. The supersaturation conditions are obviously affected also by the amount of drug and polymer in the inlet streams. Figure 1 shows that, in the selected range of operating conditions, nanoparticles with the required size (from 250 to 400 nm) can be obtained. The average diameter decreases as the inlet flow rate increases, confirming that the flow rate (and, thus, the intensity of mixing) has the influence already discussed in the literature (see for example [11,12,16,26,51,53,54]). As already discussed in our previous works and described by several research groups, such an inverse correlation between the flow rate and diameter was valid up to a flow rate threshold (in our system, approximately 50–80 mL/min); in fact, no further nanoparticle size reduction can be obtained once the mixing time is shorter than the characteristic time of the other formation processes.

**Figure 2:**
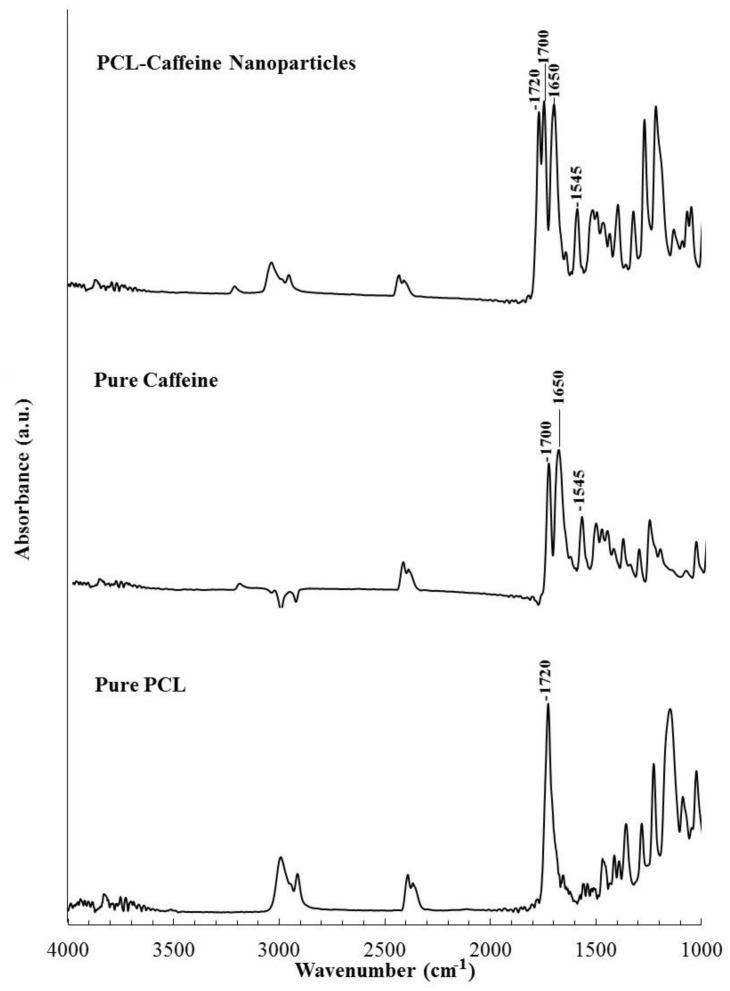
ATR-FTIR spectra of: (Bottom) PCL raw material; (Mid) caffeine; and (Top) nanoparticles with PCL-CAF_1.5_-W formulation.

By comparing the behavior of caffeine-PCL nanoparticles and unloaded PCL nanoparticles (namely, without any drug), it was observed that the addition of caffeine in the system did not alter the diameter vs flow rate trends, suggesting that the addition of a hydrophilic substance does not compromise the possibility of controlling the particle diameter by modulating the flow rate. However, for a fixed flow rate (FR), caffeine-PCL nanoparticles average size was bigger than unloaded PCL ones. This result is consistent with the incorporation of the caffeine in the polymer matrix or the adsorption of caffeine on the nanoparticle surface. To verify that incorporation really takes place, the infrared spectra of the nanoparticles were compared with those of the raw materials, i.e., PCL and caffeine. From the ATR spectra shown in Figure 2, it can be observed on the bottom that PCL shows a marked absorption peak at 1720 cm^−1^ due to the stretching of the C=O bond in the ester group while caffeine spectrum in the middle shows characteristics peaks at 1700 cm^−1^ (due to the isolated carbonyl), 1650 cm^−1^ (related to stretching of the conjugated carbonyl), and 1545 cm^−1^ (attributed to the bending of the N–H in the amide group). The spectrum of the nanoparticle powder on top of the figure shows the characteristic absorption peaks at 1720, 1700, 1650, and 1545 cm^−1^, proving that the produced nanoparticles contain both PCL and caffeine, and supporting the hypothesis that the diameter increase observed upon caffeine addition is due (at least partially) to incorporation of the drug in the particles. Figure 1 also shows that the diameter increase was much more marked when caffeine was dissolved in acetone. This observation suggests that the particles’ structure and incorporation efficiency varies according to the selection of drug solvents, as will be discussed in the next section, where effective loading will be evaluated.

### 3.2. Particles Structure

**Figure 3:**
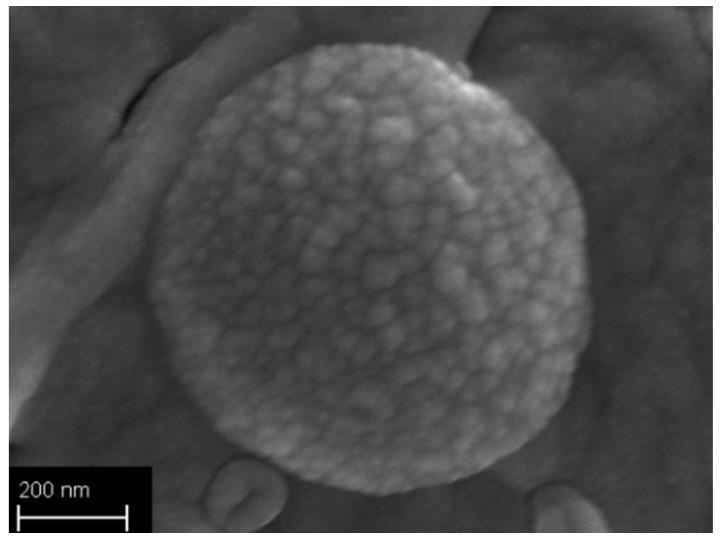
Field emission scanning electron microscopy (FESEM) image of nanoparticles (NPs) produced at 20 mL/min at 150,000× magnification for PCL-CAF_1.5_-Ac formulation.

The morphology of nanoparticles was observed by FESEM. In all cases, the particles have a spherical shape and size consistent with the one measured by DLS. In the case of nanoparticles produced by dissolving caffeine in acetone, the images evidenced a rough surface; Figure 3 shows an example obtained with PCL-CAF_1.5_-Ac formulation. The quality of the samples produced by dissolving caffeine in water was quite poor, thus not allowing definitive conclusions, even if they seemed smoother.

**Figure 4:**
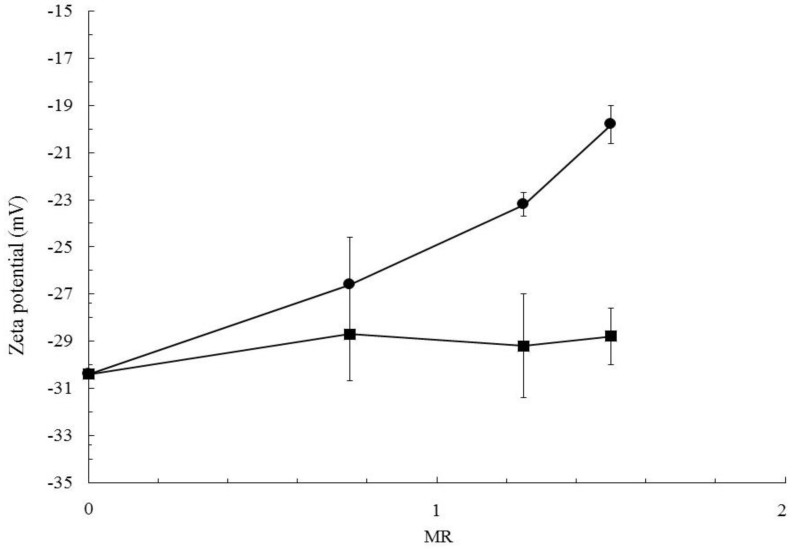
Trend of Zp with mass ratios (MR) for loaded nanoparticles obtained with caffeine dissolved in water (■) and caffeine dissolved in acetone (●). Particles were produced at FR = 20 mL/min and with initial PCL concentration of 6 mg/mL, and varying caffeine concentration to achieve the required MR.

To complete the investigation of the nanoparticle surface and give an insight into the reasons of the surface roughness, the nature of the surface charge was investigated measuring the zeta potential of the nanoparticles produced with different formulations. Samples produced under the same drug-to-polymer mass ratio, but different caffeine solvents (acetone or water), were compared. As shown in Figure 4, all formulations have a negative Zp due to the charges induced by the carbonyl groups of PCL, which oriented its polar groups toward the aqueous environment during the precipitation process. Two different trends in Zp are displayed when the amount of caffeine in the formulation is increased (keeping the PCL concentration constant at 6 mg/mL). If water is used as the solvent, Zp values do not significantly change, while for acetone Zp values remain negative, but tend to decrease in absolute value. The two different trends can be explained by considering that the caffeine molecule has nitrogen containing groups in its structure, which can be easily protonated in an aqueous environment. Therefore, caffeine can induce a positive charge if adsorbed on the particle surface. The two different Zp trends suggest that caffeine tends to be adsorbed on the particle surface when it is dissolved in acetone, thus making the surface charge less negative, while it is not adsorbed to an extent that causes an increase of Zp when it is dissolved in water.

**Figure 5:**
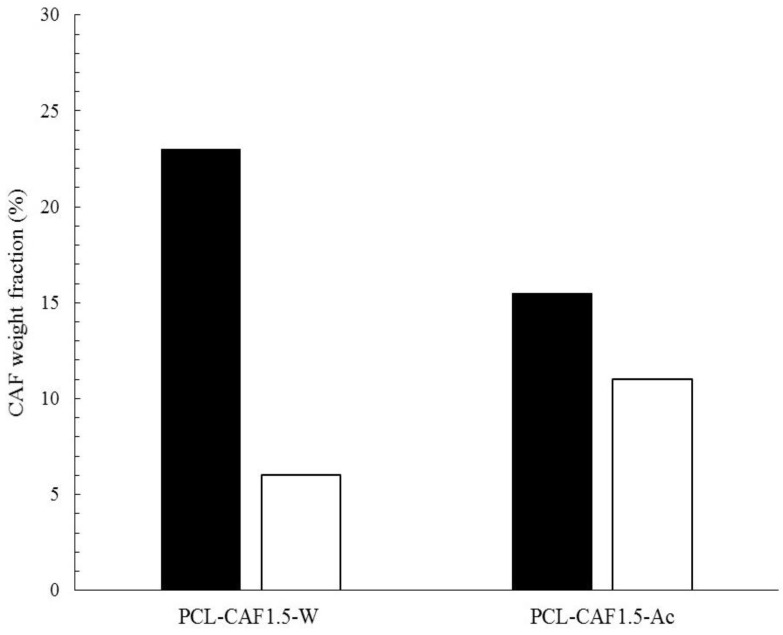
Comparison of surface weight percentage measured by XPS (white bar), with overall LC measured by UV-Vis (black bar) for samples, PCL-CAF_1.5_-W and PCL-CAF_1.5_-Ac, produced at FR = 20 mL/min.

This hypothesis was tested by conducting the XPS analysis to evidence the single elements present in the external layers of our system. The aim of the analysis was to quantify the amount of nitrogen atoms and to calculate the concentration profiles of caffeine in the particles. In fact, the XPS analysis under the etching mode allowed estimation of the caffeine weight percentage in the outer 20 nm thickness of the NPs. This quantitative analysis was carried out by monitoring the peaks in the N1s region of the spectrum (400 eV), and normalizing the peaks with the sensitivity factor of the XPS analyzer. The methodology through which the peaks were integrated and the geometry was optimized has been extensively described in our previous work [55]. The surface composition estimated by this method was then compared with the overall loading capacity to understand whether caffeine was more concentrated in the outer layer or evenly distributed in the core. The comparison among the surface and overall caffeine concentration is represented in Figure 5. By comparing two samples produced with the same composition and process parameters, but different caffeine solvent, it was observed that most of the caffeine was incorporated in the core and only a minor fraction was present in the outer 20 nm thickness when caffeine was fed in the water stream. Contrastingly, most of the drug was adsorbed or incorporated in the outer surface when caffeine was dissolved in the acetone (but the total loading of caffeine appears to be smaller than in the other case).

**Figure 6:**
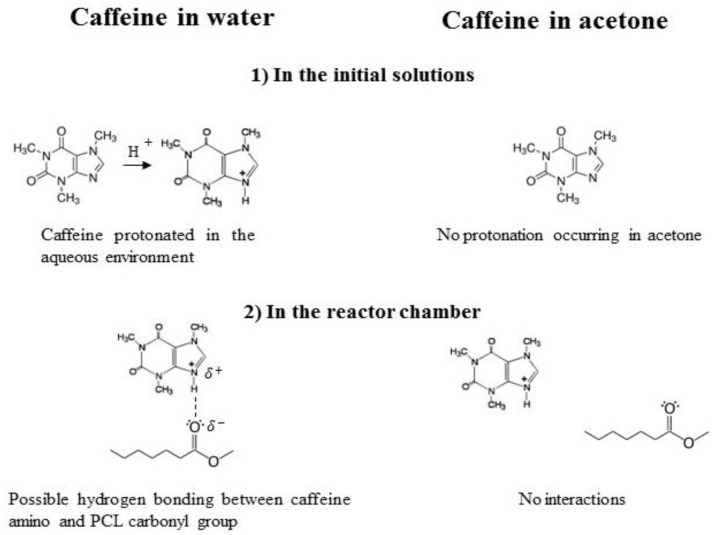

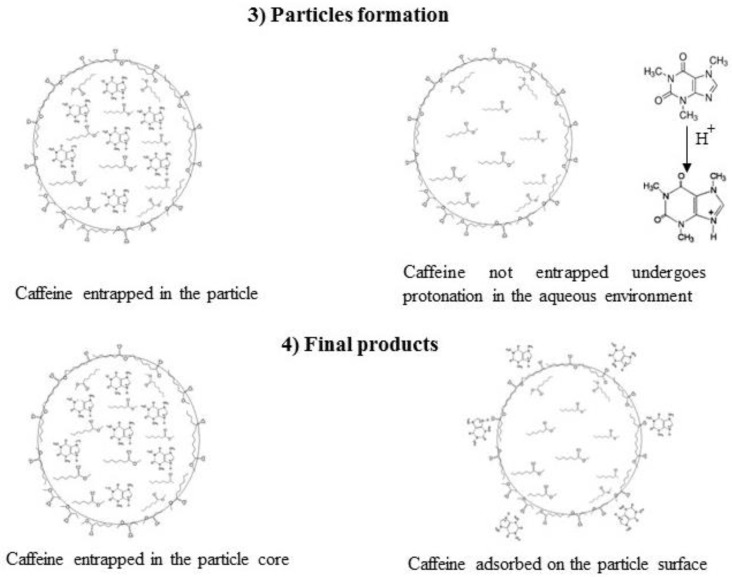
Scheme of the different encapsulation mechanisms in terms of caffeine interactions.

This result is consistent with the increase in Zp acetone-based formulations. It is clear that changing the solvent for the caffeine feed leads to a different particle structure, with a different drug distribution in the polymer shell. By solubilizing caffeine in water, it was mostly embedded in the polymer core. This result can be discussed in terms of caffeine’s chemical structure and how it interacts with the solvents and the polymer during the process. In Figure 6, the encapsulation mechanism is sketched. When caffeine is dissolved in water it tends to be protonated [56] and can therefore interact with the negatively charged carbonyl group of PCL by hydrogen bonding and Van der Waals interactions. The interactions between the oppositely charged species contribute to drug encapsulation in the polymer matrix, with a mechanism similar to the one explained by Pinkerton [57]. Contrastingly, when caffeine is dissolved in acetone, it was not protonated; moreover, it diffuses toward water upon jet collision since it is the solvent to which the drug has more affinity, while it has been shown that the environment around the PCL chains remains richer in acetone during the solvent displacement process [19,58]. Once caffeine-water interaction occurs, the protonated drug can electrostatically interact with PCL during the growth and aggregation stage. This analysis not only elucidated the particles’ structures and formation mechanism, but it also provided at least a qualitative explanation of the difference in the loading and particle size observed with the two feeds. In fact, the electrostatic interaction favors the encapsulation of caffeine, and this justifies the higher loading observed with caffeine fed in the water stream (see Figure 5). On the other hand, notwithstanding the loading is lower, a more marked increase in the NP diameter is observed when caffeine is fed in acetone (see Figure 1). The PCL-caffeine surface interaction makes the colloidal system less stable by reducing the absolute value of Zp, therefore, some aggregation phenomena may occur and cause the particles’ size to increase.

### 3.3. Drug Encapsulation and Release

**Figure 7:**
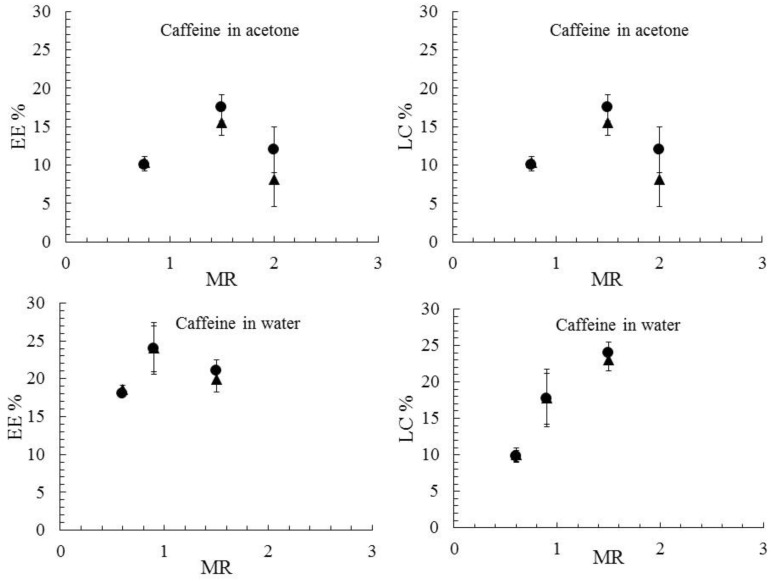
LC and EE as a function of MR for samples produced with caffeine in acetone (top) and caffeine in water (bottom). Results obtained with direct (▲) and indirect (●) protocols. All samples produced at FR = 20 mL/min.

The FNP process allowed PCL-caffeine nanoparticles with a tunable size and controlled structure to be obtained. To test the suitability of such nanocarriers in pharmaceutical applications, their capability of incorporating and slowly releasing the active principle was quantitatively evaluated. The LC and EE were calculated both by direct and indirect methods, based on the supernatant and the solid phase analysis. Trends of the LC and EE as a function of the MR for different formulations are shown in Figure 7. The consistency of the direct and indirect methods proves the effective encapsulation of the drug by the NPs system; moreover, it also means a complete recovery of caffeine, which proves that no significant degradation of the molecule occurred. EE values did not show a clear trend with the MR in both cases, but a 5%–10% higher value was observed when water was used as the solvent. This result is consistent with the literature, which showed the EE in FNP usually depends on the drug solubility in the solvent and antisolvent and not on the drug to polymer mass ratio [25,59]. The higher EE achieved in water was mainly due to the chemical affinity of protonated caffeine with PCL, as described in the previous section. The EE values were comparable with the ones achieved with other established drug encapsulation procedures [39,42,43] and these results demonstrated the suitability of FNP as an encapsulation technique for hydrophilic substances.

**Figure 8:**
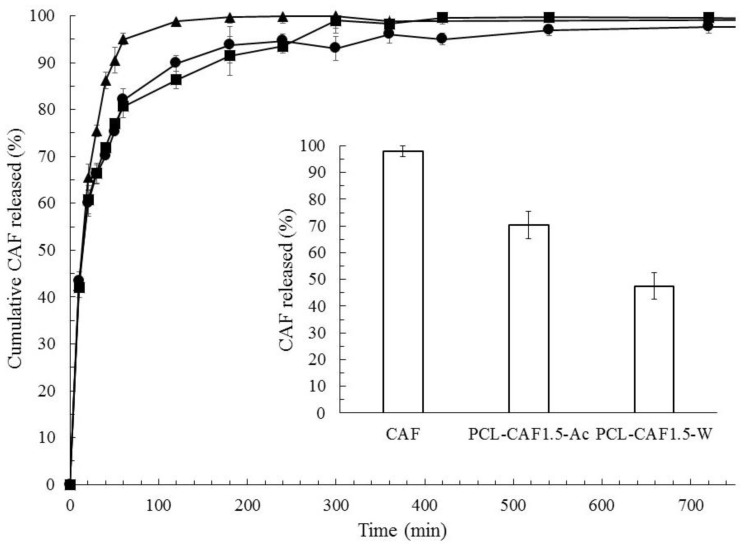
Cumulative normalized release curves for caffeine (▲), PCL-CAF_1.5_-Ac (●), and PCL-CAF_1.5_-W (■). The histogram in the inset shows the amount of caffeine released in the three tests. All NPs samples produced at FR = 20 mL/min.

As far as the LC of caffeine-in-water formulations was concerned, an increasing trend with MR was observed, suggesting that the LC can be controlled by adjusting the relative content of caffeine and PCL. In caffeine-in-acetone formulations, no particular trends of LC can be observed except that the formulation PCL-CAF_2_-Ac showed the lowest LC% and EE%. Such behavior was ascribed to the low initial PCL amount in the formulation, leading to insufficient polymer available for drug encapsulation. LC values were again larger if caffeine was dissolved in water, in accordance with the EE results. At MR 1.5, the highest LC was achieved for both water and acetone and therefore these samples were chosen for the release test. The release kinetics were studied by a dialysis test, and the cumulative release curves, shown in Figure 8, were obtained by normalizing the release data over the maximum mass of the drug found in the receptor fluid; in the histogram of the same figure, the percentage amount of drug released with respect to the initial amount encapsulated in the nanoparticles is plotted. The caffeine solution, used as a control, displayed a very fast release and the concentration peak in PBS was reached in one hour, as it occurs in the human body. The NPs systems curves instead are characterized by a smoother rise in drug release, with the plateau reached in approximately 6 h, demonstrating the capability of NPs to delay and control caffeine release. An initial burst release was noticed and was ascribed to free caffeine in the supernatant; such an amount was quickly released in less than 30 min, after which a change in the slope of the curves occurs, suggesting that the release from the nanoparticles was taking place. Concerning the total amount of caffeine released at the plateau, the control caffeine solution released the whole drug amount while the NPs formulations released only a fraction of it. These results can be explained considering the slow degradation of PCL, occurring over a long period of time (e.g., weeks) and in the presence of enzymes. The observed release was therefore only due to caffeine diffusion from the core to the NP surface without any effect of polymer erosion; as the kinetics were dominated by diffusion, the shapes of the release curves are similar for both the nanoparticles systems. The two different formulations showed instead different fractions of caffeine released with respect to the loaded amount. As a matter of fact, it can be easily forecasted that caffeine would be released more easily if located in the outer surface layers. Therefore, the PCL-CAF_1.5_-Ac, which contains about 70% of the incorporated drug in the outer 20 nm (as evidenced by XPS analysis), displayed a higher amount of drug released if compared to the PCL-CAF_1.5_-W sample; it was observed that the total amount of drug released was higher than the one found by XPS in the outer 20 nm. This fact can be explained by PCL swelling, which promotes the diffusion of the drug out of the particles.

To better elucidate the drug release mechanism, the release test data were fitted by different kinetic models. The regression coefficients for each model (*R*^2^) are reported in Table 3. Free caffeine control best fits the first order model, as expected when release is controlled by diffusion without any other resistance to mass transfer. Concerning the NPs samples, they were better fit by diffusion-based models rather than erosion based ones. The PCL-CAF_1.5_-Ac sample can be fitted either by first order or Baker and Lonsdale kinetics, even if the correlation coefficient is not very high in all the cases. Caffeine adsorbed onto the particles surface will be promptly released according to the first order model while that incorporated will be released according to Baker–Lonsdale kinetics, which describes the release from a spherical matrix. For the PCL-CAF_1.5_-W sample, the best fit was by the Baker and Lonsdale equation, as was expected given the spherical structure of the particle and the distribution of the drug in the polymer matrix.

The evaluation of drug loading and release showed that the two different NPs structures obtained by dissolving caffeine either in acetone or in water led to different functional properties: If acetone was used as the solvent, the particle displayed lower loading combined with a fast and total release, while if water was used as the solvent, the particle displayed a higher drug content and slower release of the active principle, acting indeed as reservoir systems.

## 4. Conclusions

The present work investigated the ability of FNP to encapsulate hydrophilic drugs in polymeric nanocarriers to overcome one of the major drawback of this technology. Caffeine was tested as a model drug to be encapsulated in PCL nanoparticles. The control of the particle size by tuning the inlet flow rate, one of the advantages of FNP, was maintained without any interference due to the introduction of a hydrophilic drug in the system. The type of solvent for caffeine solubilisation played a role in determining the final nanoparticles structure. On the basis of XPS data, combined with the results of LC and Zp values, it can be concluded that caffeine was preferentially located in the outer layer in the case of caffeine dissolved in acetone, while it is more embedded in the polymer matrix in the case of caffeine dissolved in water. This result was mainly ascribed to the different encapsulation mechanisms envisaged for the two configurations. Accordingly, nanoparticles produced with caffeine dissolved in water showed a higher EE and LC with respect to the ones produced with caffeine dissolved in acetone. The EE was comparable with values found in the literature for the same drug using other encapsulation techniques. This proves the suitability of the FNP technique for the encapsulation of hydrophilic substances. The release test showed a delayed delivery from the NPs systems with some peculiarities due to their different structures. If acetone was used as the solvent, the nanoparticles released most of the drug content while, if caffeine was dissolved in water, the particle did store half of the drug loaded acting as a reservoir. The research work proposed a novel application of the FNP technology, but also underlined some key issues that must be considered in designing a nanoprecipitation process to encapsulate hydrophilic substances. Firstly, the choice of the solvents is of crucial importance since this work and a previous one showed that the higher the solubility of the drug in the antisolvent substance, the higher the encapsulation efficiency. In our previous work, the successful encapsulation of melatonin [26] was mainly due to its scarce solubility in the antisolvent used. To rationally choose the polymer-drug-solvents to be used in the process, Hansen solubility parameters and the distance between them should be considered [51]. 

Another factor to be taken into consideration is the drug chemical structure. In our work, we observed how the chemical affinity between the drug and the polymer can display surface interactions. The opportunity of exploiting this in the design of the formulation could allow the surface of the nanoparticles to be functionalized to improve their performances in vivo. The present paper proposed a novel application of FNP and a simple way to produce drug loaded nanocarriers, in which the carrier structure could be changed by minor process modifications. Indeed, by dissolving caffeine in water, nanoparticles with suitable characteristics for transdermal delivery, in terms of structure, size, and LC, were obtained. For this reason, our future research will test the opportunity of using the nanoparticles produced by dissolving caffeine in water for dermatological applications, which require a hydrophobic carrier due to the hydrophobic nature of the skin’s outer layers and free caffeine would hardly be released. In such an application, the best performance of the developed carrier is expected.

## Figures and Tables

**Table 1:** Poly-ε-caprolactone (PCL)-caffeine nanoparticles’ formulations for which loading capacity (LC) and encapsulation efficiency (EE) were measured.

Sample Label	*C*_PCL_ (mg/mL)	C_CAF_ (mg/mL)	CAF Solvent	MR
PCL-CAF_0.76_-Ac	10.0	7.6	Acetone	0.76
PCL-CAF_1.5_-Ac	6.0	9.0	Acetone	1.50
PCL-CAF_2_-Ac	4.5	9.0	Acetone	2.00
PCL-CAF_0.6_-W	15.0	9.0	Water	0.60
PCL-CAF_0.9_-W	10.0	9.0	Water	0.90
PCL-CAF_1.5_-W	6.0	9.0	Water	1.50

**Table 2:** PCL-caffeine nanoparticles formulations for which the zeta potential was measured.

Sample Label	*C*_PCL_ (mg/mL)	*C*_CAF_ (mg/mL)	CAF Solvent	MR
Pure PCL	6.0	0.0	-	0.00
PCL-CAF_0.75_-Ac	6.0	4.5	Acetone	0.75
PCL-CAF_1.25_-Ac	6.0	7.5	Acetone	1.25
PCL-CAF_1.5_-Ac	6.0	9.0	Acetone	1.50
PCL-CAF_0.75_-W	6.0	4.5	Water	0.75
PCL-CAF_1.25_-W	6.0	7.5	Water	1.25
PCL-CAF_1.5_-W	6.0	9.0	Water	1.50

**Table 3:** Correlation coefficient for fitting of the release curves with different kinetic models.

Sample	Zero Order	First Order	Higuchi	Hixon Crowell	Baker Lonsdale
CAF Control	0.14	0.90	0.60	0.48	0.70
PCL-CAF_1.5_-Ac	0.19	0.85	0.84	0.69	0.89
PCL-CAF_1.5_-W	0.07	0.94	0.84	0.84	0.99
